# Comparable dynamic cerebral autoregulation and neurovascular coupling of the posterior cerebral artery between healthy men and women

**DOI:** 10.1111/cns.14584

**Published:** 2024-02-08

**Authors:** Hongxiu Chen, Liuping Cui, Songwei Chen, Ran Liu, Xijuan Pan, Fubo Zhou, Yingqi Xing

**Affiliations:** ^1^ Department of Vascular Ultrasonography Xuanwu Hospital, Capital Medical University Beijing China; ^2^ Beijing Diagnostic Center of Vascular Ultrasound Beijing China; ^3^ Center of Vascular Ultrasonography, Beijing Institute of Brain Disorders, Collaborative Innovation Center for Brain Disorders Capital Medical University Beijing China

**Keywords:** cerebral blood flow velocity, dynamic cerebral autoregulation, mean arterial pressure, neurovascular coupling, transcranial doppler

## Abstract

**Aims:**

Most studies focus on dynamic cerebral autoregulation (dCA) in the middle cerebral artery (MCA), and few studies investigated neurovascular coupling (NVC) and dCA in the posterior cerebral artery (PCA). We investigated NVC and dCA of the PCA in healthy volunteers to identify sex differences.

**Methods:**

Thirty men and 30 age‐matched women completed dCA and NCV assessments. The cerebral blood flow velocity (CBFV) and mean arterial pressure were evaluated using transcranial Doppler ultrasound and a servo‐controlled plethysmograph, respectively. The dCA parameters were analyzed using transfer function analysis. The NCV was evaluated by eyes‐open and eyes‐closed (24 s each) periodically based on voice prompts. The eyes‐open visual stimulation comprised silent reading of Beijing‐related tourist information.

**Results:**

The PCA gain was lower than that of the MCA in all frequency ranges (all *p* < 0.05). Phase was consistent across the cerebrovascular territories. The cerebrovascular conductance index (CVCi) and mean CBFV (MV) of the PCA were significantly higher during the eyes‐open than eyes‐closed period (CVCi: 0.50 ± 0.12 vs. 0.38 ± 0.10; MV: 42.89 ± 8.49 vs. 32.98 ± 7.25, both *p* < 0.001). The PCA dCA and NVC were similar between the sexes.

**Conclusion:**

We assessed two major mechanisms that maintain cerebral hemodynamic stability in healthy men and women. The visual stimulation‐evoked CBFV of the PCA was significantly increased compared to that during rest, confirming the activation of NVC. Men and women have similar functions in PCA dCA and NCV.

## Graphical abstract

This study explored sex‐differences in dynamic cerebral autoregulation (dCA) and neurovascular coupling (NVC) of the posterior cerebral artery (PCA) between healthy men and women. We observed a significant increase in the visual stimulation‐evoked cerebral blood flow velocity (CBFV) of the PCA compared to that during rest, confirming activation of NVC.

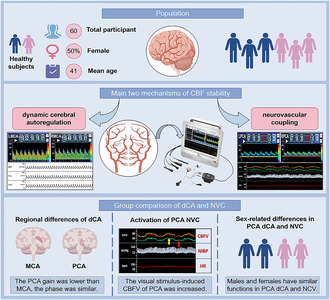

## 1 INTRODUCTION

Cerebral autoregulation (CA) is defined as the ability to adjust the caliber of small cerebral vessels to change vascular resistance and maintain a relatively constant cerebral blood flow (CBF) when the average arterial pressure fluctuates.^1^ It is also an important method for preventing over‐ or underperfusion of brain tissue. CA can be divided into two main types: static CA (sCA) and dynamic cerebral autoregulation (dCA). The former refers to changes in CBF before and after a slow change in blood pressure (BP), reflecting the overall regulatory ability of the brain and its upper and lower regulatory limits. Due to its inability to reflect the impact of BP fluctuations on CA capacity over a short time and the frequent need for pharmacological interventions to change BP, sCA has limited clinical application.^1^, ^2^ Currently, more researchers are focused on dCA, which describes the immediate response of CBF to rapid BP changes. In 1982, with the advent of transcranial Doppler (TCD) ultrasound, the sustainable measurement of cerebral blood flow velocity (CBFV) brought about a revolution in the field of CA, achieving real‐time hemodynamics analysis.^3^ The TCD has become the most widely used technology for evaluating the dCA function.^4^

With the progress and development of dCA analysis technology, numerous studies have shown that impaired dCA is strongly associated with many neurological diseases, such as ischemic stroke, cognitive impairment, cerebral small vessel disease, and brain injury.^5^, ^6^, ^7^ Therefore, reliable assessment of dCA may facilitate the understanding of clinical severity and guide personalized patient care. However, most previous studies on regional dCA have focused on the middle cerebral artery (MCA), ignoring potential regional heterogeneity in blood vessels. Indeed, anatomical differences exist between the anterior and posterior circulation and in sympathetic nerve activity, cerebrovascular reactivity, and CBF.^8^, ^9^, ^10^ A recent review by Koep et al. suggested that future research should focus on distinguishing regional differences in dCA.^9^ Notably, neurovascular coupling (NVC), one of the main mechanisms affecting cerebral hemodynamics, is a phenomenon in which the body changes regional CBF according to neural activity and metabolism.^11^

While the effects of sex on CBFV, cerebrovascular reactivity to carbon dioxide, and dCA of the MCA have been extensively reported,^12^, ^13^ less attention has been paid to the effect of sex on dCA of the posterior cerebral artery (PCA) in the supine position using transfer function analysis (TFA). The TFA utilizes a fast Fourier decomposition of stationary input and output signals to decompose them into the sum of sines and cosines of multiple frequencies.^14^ Of note, the 2022 updated white paper of the cerebrovascular research network (CARNet) proposed that a dCA study design requires a balanced number of male and female participants, considering the impact of sex on the outcomes.^14^

Consequently, the present study explored dCA of the PCA using TFA methods and NVC and analyzed sex differences in dCA in healthy individuals to provide a basis for future research on dCA impairment. If there are sex differences in dCA, this may explain sex differences in, for example, patients with migraine.

## 2 METHODS

### 2.1 Study design and participants

Healthy volunteers were consecutively recruited from the Capital Medical University Xuanwu Hospital between June 2022 and November 2022. This study was approved by the Ethics Committee of the Capital Medical University Xuanwu Hospital (approval number: [2021]109).  Individuals were eligible if they were ≥18 years old. Exclusion criteria were as follows: the history of cardiovascular and cerebrovascular disease, hypertension, diabetes, or any conditions known to affect dCA or NVC (such as chronic psychiatric diseases, dementia, carotid and intracranial artery stenosis) and poor or closed bilateral temporal windows. We collected the relevant data, such as age, sex, heart rate (HR), BP, CBFV, and dCA parameters from all study participants.

### 2.2 Assessment of the dCA

dCA assessments were performed by specialized vascular ultrasound physicians based on the recommendations of the International White Paper from CARNet.^14^ The examination of subjects was performed in a temperature‐controlled environment of 22–24°C. Before measurements, participants had to (1) avoid alcohol, chocolate, and caffeine for at least 12 h; (2) refrain from moderate (or more intense) exercise for ≥6 h; and (3) avoid eating high‐calorie food for at least 4 h. The participants rested for 15 min without crossing their legs before the dCA assessment, which was performed using a TCD (EMS‐9D Pro; Delica Medical, Shenzhen, China). The CBFV of the MCA at a depth of 50–65 mm and of the PCA at 60–70 mm were measured using a 1.6‐MHz probe in the bilateral temporal windows. Noninvasive continuous beat‐to‐beat BP (NIBP) was recorded using a finger photoplethysmograph sensor (Finometer®, Arnhem, The Netherlands). Before NIBP measurement, the BP was measured at the brachial artery using an Omron sphygmomanometer to correct baseline BP. The sampling frequency of the Doppler trace and NIBP signal was 125 Hz. In addition, a nasal cannula was connected to monitor end‐tidal carbon dioxide (Et‐CO_2_). Continuous CBFV from the MCA and PCA, NIBP, HR, and Et‐CO_2_ were recorded in real time with the patient in the supine position for 10 min.

### 2.3 Neurovascular coupling

The NVC of the PCA is mainly achieved through reliable and repetitive reading of visual stimulation, which is associated with perfusion in the occipital visual cortex. Participants were assessed during a 2‐min baseline period with eyes‐closed and six repeated cycles of 24 s with eyes‐open (silent reading–visual stimulation) followed by 24 s with eyes‐closed. The visual stimulus consisted of reading material in Chinese related to general tourist information about Beijing. The mean CBFV (MV) of the PCA and mean arterial pressure (MAP) at rest and during each cycle during silent reading were measured, and cerebrovascular conductance index (CVCi) = PCA MV/MAP,^15^ and visually evoked flow response (VEFR) = (MV‐silent reading – MV‐rest)/MV‐rest × 100% were calculated.^11^

### 2.4 Data analysis of dCA

In accordance with the recommendations of CARNet, we selected stable 5‐min NIBP and TCD monitoring data from the recordings for dCA analysis with a Hanning window of 100 s in length and a 50% overlap. The TFA method was used to quantify the dCA parameters, including gain, phase, and coherence at very low frequency (VLF, 0.02–0.07 Hz), low frequency (LF, 0.07–0.20 Hz), and high frequency (HF, 0.2–0.50 Hz).^14^ The average values of the dCA parameters in both hemispheres were used for further analyses. Phase reflects the temporal relationship between the BP (input signals) and CBFV (output signals), whereas the gain represents the amplitude at the same frequency. Coherence approaching 1.0 reflects a linear relationship between oscillations in BP and CBFV. Among them, a lower phase and higher gain reflect impaired dCA, and the phase is more reliable than other TFA parameters. If coherence is <0.5, the linearity condition may be violated in this frequency range.^16^ Thus, only dCA parameters with a coherence ≥0.5 were analyzed in the present study. Regarding the frequency domain, the VLF and LF indices were of concern for dCA.

### 2.5 Statistical analyses

All statistical analyses were performed using IBM SPSS software (version 27.0; Armonk, NY, USA). All continuous data were assessed for normal distribution using Kolmogorov–Smirnov tests. Normally distributed continuous variables are expressed as means ± standard deviations and were compared using *t*‐tests. In contrast, non‐normally distributed data are presented as median with interquartile ranges and were compared using the Mann–Whitney *U*‐test. Categorical variables are expressed as percentages and were analyzed using the chi‐square test. Group differences in MAP and MV of the PCA between resting and silent reading states were compared using a paired *t*‐test. All statistical tests were two‐sided, and statistical significance was set at *p* < 0.05.

## 3 RESULTS

### 3.1 Demographical data

Sixty healthy volunteers were enrolled in the study, including 30 men and 30 age‐matched women who completed the dCA and NVC assessments of the PCA. As shown in Table 1, the baseline MAP was higher in men than in women, and no significant differences in terms of age, body mass index, HR, and ET‐CO_2_ were found between the two groups.

**TABLE 1:** Demographic characteristics of participants.

Varibles	Male (*n* = 30)	Female (*n* = 30)	*p*
Age (y)	40.83 ± 14.27	41.43 ± 12.21	0.862
BMI	23.80 ± 3.08	22.33 ± 3.64	0.073
MAP (mmHg)	90.76 ± 9.60	84.61 ± 10.83	0.024
Heart rate (beats min^−1^)	66.67 ± 8.99	66.87 ± 7.74	0.927
Et‐CO_2_	38.80 ± 2.32	38.38 ± 2.82	0.534

*Note*: Data are presented as mean ± SD.

Abbreviations: BMI, body mass index; Et‐CO_2_, end‐tidal carbon dioxide; MAP, mean arterial blood pressure.

### 3.2 Comparison of the CBFV and dCA data between the MCA and PCA

The parameters of the CBFV and dCA data for the MCA and PCA are presented in Table 2. Among them, the MV of the MCA was higher than that of the PCA in the posterior circulation (60.68 ± 10.24 vs. 32.98 ± 7.25, *p* < 0.001). In terms of the dCA parameters, the PCA gain was lower than that of the MCA in all frequency domains (PCA vs. MCA, VLF: 0.62 ± 0.24 vs. 0.76 ± 0.21; LF: 0.68 ± 0.19 vs. 0.99 ± 0.22; HF: 0.71 ± 0.15 vs. 0.97 ± 0.22, all *p* < 0.001). The coherence of the PCA was also lower than that of the MCA in the LF and HF range (both *p <* 0.05). Phase was not statistically significantly different between groups.

**TABLE 2:** Comparison of dCA parameters between the middle cerebral artery and the posterior cerebral artery.

Varibles	MCA (*n* = 60)	PCA (*n* = 60)	*p*
MV (cm s^−1^)	60.68 ± 10.24	32.98 ± 7.25	<0.001
VLF (0.02–0.07 Hz)			
Gain (cm s^−1^ mmHg^−1^)	0.76 ± 0.21	0.62 ± 0.24	<0.001
Phase (degree)	69.74 ± 19.71	66.81 ± 23.75	0.359
Coherence	0.65 ± 0.07	0.64 ± 0.06	0.152
LF (0.07–0.2 Hz)			
Gain (cm s^−1^ mmHg^−1^)	0.99 ± 0.22	0.68 ± 0.19	<0.001
Phase (degree)	44.93 ± 11.85	44.75 ± 14.34	0.915
Coherence	0.72 ± 0.08	0.69 ± 0.07	0.002
HF (0.2–0.5 Hz)			
Gain (cm s^−1^ mmHg^−1^)	0.97 ± 0.22	0.71 ± 0.15	<0.001
Phase (degree)	12.12 ± 10.12	12.60 ± 11.96	0.748
Coherence	0.75 ± 0.06	0.73 ± 0.05	0.008

Abbreviations: HF, high frequency; LF, low frequency; MCA, middle cerebral artery; MV, Mean cerebral blood flow velocity; PCA, posterior cerebral artery; VLF, very low frequency.

### 3.3 NVC measured by visual stimulation‐evoked PCA MV and CVCi

**FIGURE 1:**
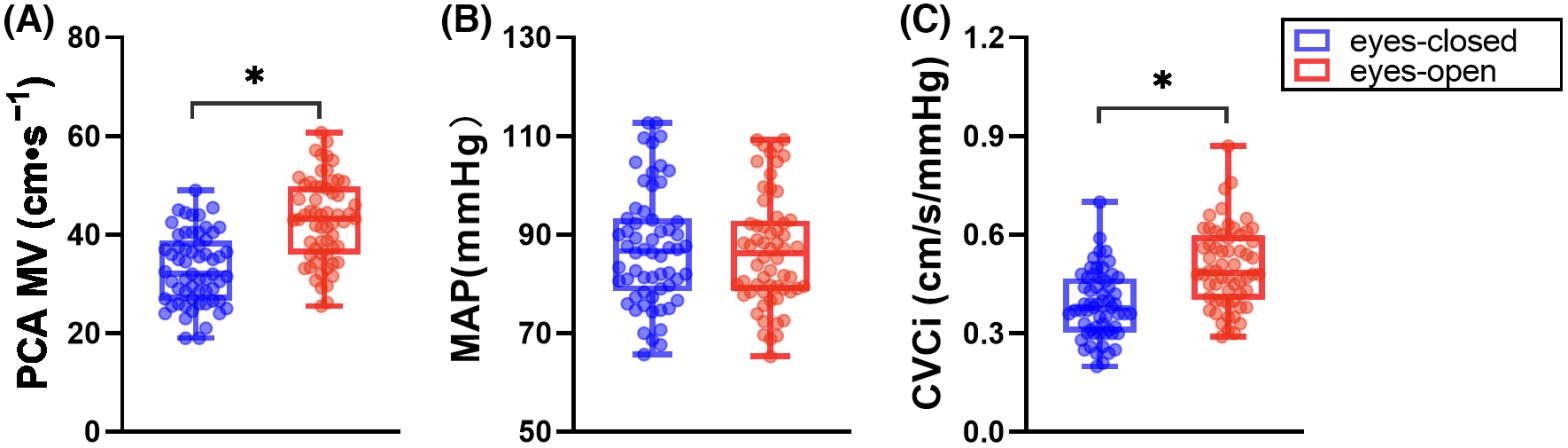
Comparison of NVC parameters between eyes‐closed (resting) and eyes‐open (silent reading). (A) The mean cerebral blood flow velocity (MV) and (C) the cerebrovascular conductance index (CVCi) of the posterior cerebral artery (PCA) were both significantly higher during the eyes‐open than the eyes‐closed period. (B) The mean arterial pressure (MAP) was similar between the eyes‐open and eyes‐closed conditions. *p < 0.05.

**FIGURE 2:**
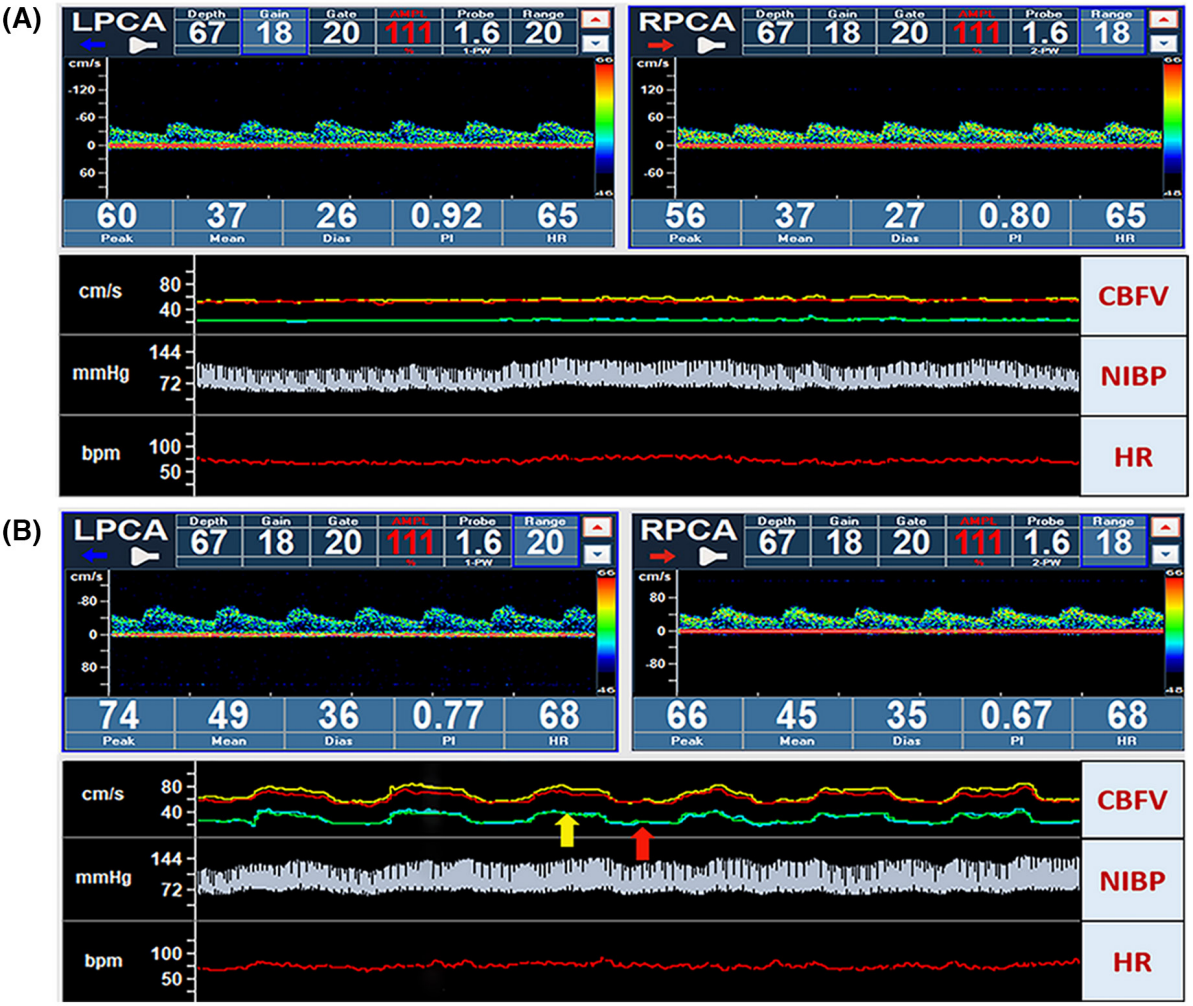
Example of volunteers performing NVC while simultaneously monitoring cerebral blood flow velocity (CBFV) and non‐invasive continuous beat‐to‐beat blood pressure (NIBP). (A) CBFV and NIBP during the 2‐min baseline period with eyes closed. (B) During periodic silent reading with eyes open, CBFV increased (yellow arrow) compared to when the eyes were closed (red arrow) and baseline period, while NIBP remained stable.

Regarding NCV, the MAP was similar between the eyes‐open and eyes‐closed conditions. In contrast, PCA MV and CVCi were higher during visual stimulation (eyes‐open) than when eyes were closed (both *p* < 0.001, Figure 1 and Table 3). The CBFV of the PCA was higher during silent reading than when the participants were at rest or had their eyes closed (Figure 2).

**TABLE 3:** Comparison of NVC parameters between resting and silent reading.

Varibles	Eyes‐closed (resting)	Eyes‐open (silent reading)	*p*
PCA MV (cm s^−1^)	32.98 ± 7.25	42.89 ± 8.49	<0.001
MAP (mmHg)	87.26 ± 11.76	86.81 ± 11.43	0.286
CVCi (cm s^−1^ mmHg^−1^)	0.38 ± 0.10	0.50 ± 0.12	<0.001

Abbreviations: CVCi, index of the cerebrovascular conductance; MAP, mean arterial pressure; PCA MV, mean blood flow velocity of the posterior cerebral artery.

### 3.4 A comparison of PCA dCA and NCV between men and women

**FIGURE 3:**
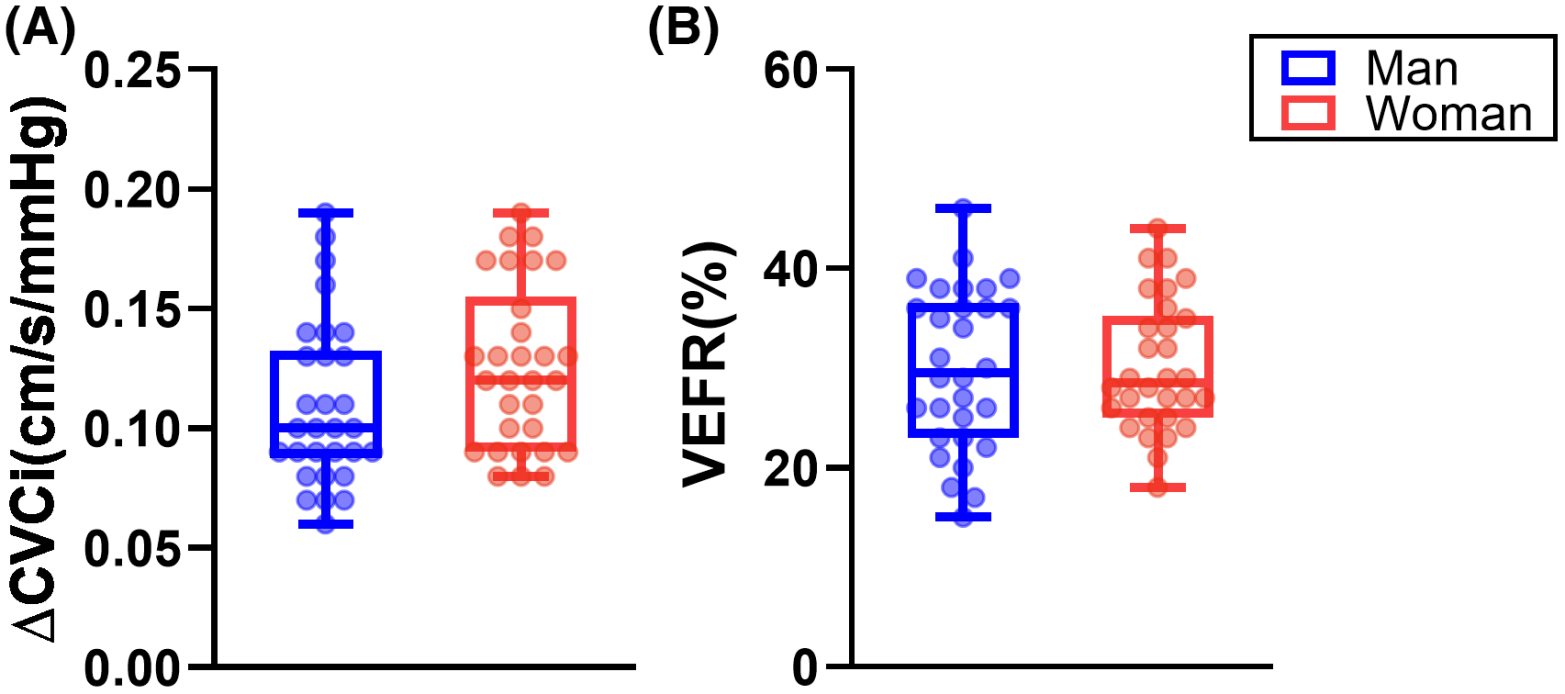
Comparison of NVC response parameters in age‐matched men and women. There was no significant sex difference with respect to (A) change in the cerebrovascular conductance index (∆CVCi) and (B) visually evoked flow response (VEFR).

As shown in Table 4, 30 women and 30 age‐matched men underwent dCA assessment at the PCA. No difference between the sexes was found with respect to PCA gain, phase, or coherence for all frequency ranges. ∆CVCi (men vs. women: 0.10 [0.08–0.13] vs. 0.12 [0.09–0.15], *p* = 0.065) and VEFR (%) (men vs. women: 29.5 [23.0–36.5] vs. 28.5 [25.0–35.2], *p* = 0.965) for the sexes are shown in Figure 3.

**TABLE 4:** Comparison of dCA parameters of PCA between different genders.

Varibles	Man (*n* = 30)	Woman (*n* = 30)	*p*
PCA MV(cm s^−1^)	31.70 ± 7.48	34.26 ± 6.90	0.172
VLF (0.02–0.07 Hz)			
Gain (cm s^−1^ mmHg^−1^)	0.59 (0.40, 0.81)	0.54 (0.49, 0.79)	0.695
Phase (degree)	65.90 ± 25.76	67.72 ± 21.96	0.769
Coherence	0.65 ± 0.06	0.63 ± 0.05	0.076
LF (0.07–0.2 Hz)			
Gain (cm s^−1^ mmHg^−1^)	0.64 ± 0.19	0.72 ± 0.19	0.143
Phase (degree)	45.07 ± 13.32	44.44 ± 15.51	0.867
Coherence	0.69 ± 0.07	0.68 ± 0.07	0.540
HF (0.2–0.5 Hz)			
Gain (cm s^−1^ mmHg^−1^)	0.69 ± 0.11	0.73 ± 0.17	0.242
Phase (degree)	14.05 ± 11.58	11.14 ± 12.42	0.349
Coherence	0.73 ± 0.04	0.73 ± 0.05	0.497

Abbreviations: HF, high frequency; LF, low frequency; PCA MV, mean cerebral blood flow velocity of posterior cerebral artery; VLF, very low frequency.

## 4 DISCUSSION

The present study investigated the two main mechanisms ensuring appropriate CBF in the PCA: dCA and NVC. We observed a significant increase in the visual stimulation evoked MV of the PCA compared to that during rest, confirming activation of NVC. And our study demonstrated that dCA (i.e., the gain, phase and coherence) and NVC response parameters (i.e., ∆CVCi and VEFR) for the PCA were similar between healthy men and women. Besides, we further explored whether regional differences in the dCA exist between the posterior and anterior circulation, and the results revealed that gain was lower in the PCA than in the MCA, indicative of a more effective hemodynamic autoregulation. In contrast, phase was similar in both the MCA and PCA.

As dCA is a highly complex mechanism, a great deal of research has been dedicated to methods for its noninvasive evaluation, such as multimodal pressure‐flow analysis, correlation coefficient analysis, and the autoregulatory index (TFA).^17^, ^18^ The TFA method uses a fast Fourier transform to decompose a stationary input signal (BP) and output signal (CBF/CBFV) and further quantifies dCA function. In 2016, a white paper from CARNet proposed standardization of the parameters and settings of the TFA method, which has become the most widely applied method for studying dCA.^14^, ^17^ This study used TFA methods to evaluate dCA parameters of the anterior and posterior circulations. To date, most human studies on dCA have concentrated on sympathetic control of the anterior circulation. It is worth noting that there is vascular regional heterogeneity in both healthy populations and in many focal diseases such as acute ischemic stroke and cerebral hemorrhage, neglecting the attention to the dCA in PCA.^9^ At present, although some studies have focused on the difference in dCA between the anterior and posterior circulations, the results have not been consistent.^8^, ^19^, ^20^, ^21^ A recent study found that the dCA functions of both the MCA and PCA are similar in the cold pressor test,^8^ while Reehal et al. and Labrecque et al. reported a smaller absolute gain in the PCA than in the anterior circulation in squat‐standing and spontaneous measures, respectively, suggesting that the posterior circulation may demonstrate more effective dCA,^19^, ^20^ consistent with our findings. This may be related to baseline differences in CBFV.^22^ Current evidence suggests that differences in dCA between brain regions are physiologically plausible because these heterogeneities may reflect different sympathetic and parasympathetic innervation in the anterior and posterior circulation. At the same time, different functional and anatomical features also exist between these two territories.^9^, ^23^ In their review, Koep et al.^9^ also highlighted the need for more studies to gain a better understanding of the regional heterogeneity of dCA by assessing global and regional CBF in the future.

Numerous studies have reported sex differences in dCA of the MCA.^24^, ^25^ Favre et al.^25^ showed that there was no difference in dCA between men and women in the supine posture. However, there are contrasting data showing that dCA function in men is better than that in women in the supine posture.^26^ While the PCA is an integral part of the cerebrovasculature, it remains poorly understood whether sex has an effect on dCA in the posterior circulation. Currently, similar to the MCA, the literature regarding the effect of sex on dCA in the PCA shows inconsistent results.^21^, ^27^, ^28^ Vavilala et al.^27^ observed that girls had a higher autoregulatory index (ARI) in the basilar artery than healthy boys (a higher ARI indicates greater dCA). Conversely, Tontisirin et al. similarly applied ARI to quantify autoregulatory functions and found no sex differences in dCA of the MCA or basilar arteries in prepubertal children.^28^ Similar to our results, a recent study, which also used the TFA method to assess dCA in healthy subjects, demonstrated that men and women have similar dCA in the PCA in terms of spontaneous oscillations in BP.^21^ They further assessed dCA driven by squat–stand maneuvers and reached the same conclusion.^21^ Thus, further investigations are required given the differences in hormone levels between the sexes and the different methods of evaluating dCA.

NVC, the coupling between local neuronal activity and changes in CBF, causes an increase in regional CBF to meet metabolic demands, mainly through a series of regulatory activities by cells such as astrocytes.^15^ TCD, with its high temporal resolution and non‐invasive nature, can not only record CBFV but can also monitor the changes and responses of the vascular system induced by internal or external stimuli. It has become a commonly used and useful tool for evaluating NVC.^11^ According to published NVC guidelines, the NVC response assessment protocol is mainly based on visual stimulation (i.e., reading with eyes open, flashing screens), which has been previously reported to be associated with visual cortical perfusion.^15^ During silent reading with eyes open, we observed a marked elevation in CBFV in the PCA, which is closely associated with Brodmann areas 17, 18, and 19 in the visual occipital cortex, which may be selectively activated.^29^ Additionally, CBFV can be increased by releasing relevant neurotransmitters and transmitting vascular activity signals.^29^, ^30^ Previous studies have shown that NVC is impaired in clinical populations, such as those with high‐level spinal cord injury and stroke.^31^, ^32^ This study focused on the normative values of NVC in healthy humans, which can provide a reliable basis for future research to determine the impairment of NVC. Moreover, we further explored whether sex differences can confound NVC assessment, as this is not well studied. The existing literature, consistent with our application of a similar methodology and TCD assessment of NVC, also revealed that men and women have similar response magnitudes for NVC.^33^, ^34^ Interestingly, a recent study by Leacy et al. showed that the time from visual stimulation to the peak NVC response is shorter in women and that differences in NVC response integration speed may be due to sex‐related differences in brain network connectivity.^33^, ^35^ This should be studied further in the future.

Finally, this study had certain limitations. First, the current study is based on the assumptions that the intracranial artery diameter remains constant and that TCD mainly measures CBFV rather than CBF, and then further quantifies the dCA and NVC. Previous studies found that the diameter of the artery did not change significantly in response to physiological stimulation. Therefore, CBFV can be used as a reliable surrogate for CBF.^36^ In addition, given the single‐center study design with a relatively small sample size, future studies with larger sample sizes are needed to improve our understanding of dCA and NVC.

## 5 CONCLUSION

This study explored regional heterogeneity of the anterior and posterior circulation dCA. We showed that the main vessels within the cerebrovascular system (MCA and PCA) have similar phase, which quantifies the time course of hemodynamic regulation. However, the amplitude of dCA (i.e., gain) was lower in the PCA, indicative of a tighter hemodynamic autoregulation. Furthermore, we found that sex did not affect dCA and NVC responses in the PCA in healthy participants. Our study provides reliable normative values for dCA and NVC for the PCA in healthy men and women, forming a basis for further research.

## AUTHOR CONTRIBUTIONS

YX and HC conceived and designed the study. HC, SC, LC, RL, and XP contributed to the dCA and NVC data collection and methodology. HC and FZ performed the statistical analyses. HC wrote the manuscript. Prof. YX edited and critically revised the manuscript. Each authors read and approved the final version of the manuscript.

## FUNDING INFORMATION

This study was funded by the National Natural Science Foundation cultivation project of Xuanwu Hospital (No. QNPY2022007).

## CONFLICT OF INTEREST STATEMENT

The authors report that there are no conflict of interest.

## PATIENT CONSENT STATEMENT

All procedures were verbally explained in detail to the volunteers, and written informed consent was obtained.

## Data Availability

The data that support the findings of this study are available from the corresponding author upon reasonable request.
